# Supplementary material for: Atomic-scale visualization of chiral charge density wave superlattices and their reversible switching
Source: Nat Commun. 2022 Apr 5;13:1843. doi: 10.1038/s41467-022-29548-2 (PMC8983771; doi:10.1038/s41467-022-29548-2)
Supplement: Supplementary file 1 — Supplementary Information [file 41467_2022_29548_MOESM1_ESM.pdf]

Supplementary Information

Atomic-Scale Visualization of Chiral Charge Density Wave Superlattices and Their Reversible Switching

Xuan Song[1#], Liwei Liu[1#]*, Yaoyao Chen[1], Han Yang[1], Zeping Huang[1], Baofei Hou[1], Yanhui Hou[1], Xu Han[1], Huixia Yang[1], Quanzhen Zhang[1], Teng Zhang[1], Jiadong Zhou[1], Yuan Huang[1], Yu Zhang[1], Hong-Jun Gao[2] and Yeliang Wang[1]*

[1]School of Integrated Circuits and Electronics, MIIT Key Laboratory for Low-Dimensional Quantum Structure and Devices, Beijing Institute of Technology, Beijing 100081, China.

[2]Institute of Physics, Chinese Academy of Sciences, Beijing 100190, China.

*Corresponding author. Email: liwei.liu@bit.edu.cn (L.W.L.); yeliang.wang@bit.edu.cn (Y.L.W.)

1. Supplementary Note. Determination of the distance between the tip and the substrate.

2. Supplementary Figures 1 to 5

Supplementary Fig. 1. STM images and atomic models of the moving boundary of chiral domains.

Supplementary Fig. 2. Time-lapse STM images showing the movement of the chiral CDW domain boundary.

Supplementary Fig. 3. Fast Fourier transform (FFT) of chiral CDW domains.

Supplementary Fig. 4. The dynamic characterization of the chiral domain transition.

Supplementary Fig. 5. Tiny contraction (~30 pm) of the Se atomic lattice in the CDW superlattice.

Supplementary Fig. 6. Additional Bragg spots from BLG by including wide area.

Supplementary Fig. 7. The energy barrier model of the L and R chirality.

3. Supplementary Movie S1. Dynamic example of the DB movement of the chiral CDW superlattice.

4. References

1. Supplementary Note:

Determination of the distance between the tip and the substrate

For the determination of the initial tip–sample distance (V_B = -1.5 V, I_t = 20 pA), the tunneling gap is set and the STM tip (scanner) retraction or extension is recorded. Subsequently, the feedback loop is turned off and the tip is moved toward the substrate surface gently until a sudden increase in the tunneling current to a saturation of 10 nA is reached. The distance traveled during this process was 0.1 nm, which was used as the initial distance between the tip and the substrate.

2. Supplementary Figures 1 to 5

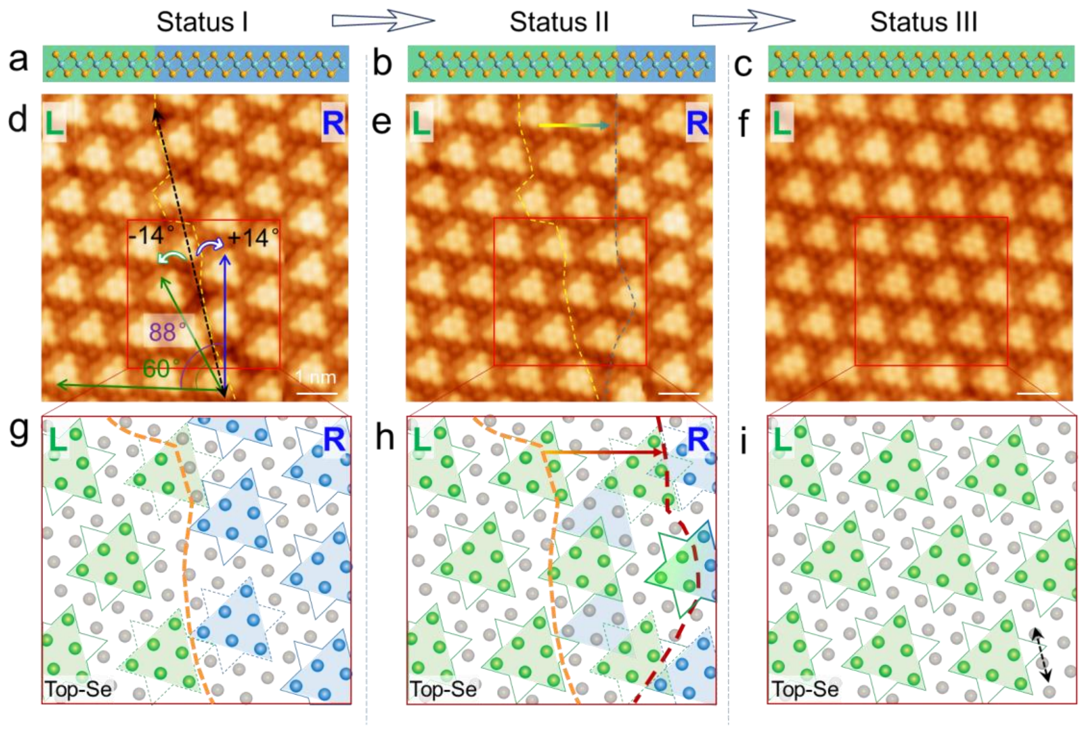

Supplementary Fig. 1. STM images and atomic models of the moving boundary of chiral domains. a-c, The side view models of the SL 1T-NbSe$_2$ with left and right chiralities, corresponding to STM images in **d-f**. The close-packed directions of different chiral SOD domains are rotated by about ±14 °relative to the close-packed direction of the Se atomic lattice, as shown in the green, blue and black arrows in (**d**) The neighboring close-packed direction of the SOD superlattice has a rotation of 60 ° angle, due to the six-fold symmetry. So, the angle between two different chiral domains can also be defined as either 28 °or 88 °, that is, 14 °-(-14 °)

= 28 ° and 14 ° -(-14 °) + 60 ° = 88 °. The rotation of the two chiral CDW domains is close to 90 °, and this observation can be found in Fig. 3 and Fig. 2d-i in the main text. **g-i**, Atomic models of SL 1T-$NbSe_2$ with the shifted CDW domain boundary (DB), describing the same area marked by the red frame in **d-f**. The green and blue balls represent Se atoms in the SOD with left and right chiralities, respectively, while the gray balls show the Se atoms surrounding the SOD. All Se atoms (in blue, green, and grey) share the same periodic hexagonal lattice.

Although there are two chiral charge density wave (CDW) domains, the atomic lattice in the top Se layer is continuous because no atomic defects are visible. The rotation of the two chiral CDW domains was approximately 28°, which can be determined by both the STM images and fast Fourier transform (FFT) images (The magnified FFT images are shown in Fig. S3).

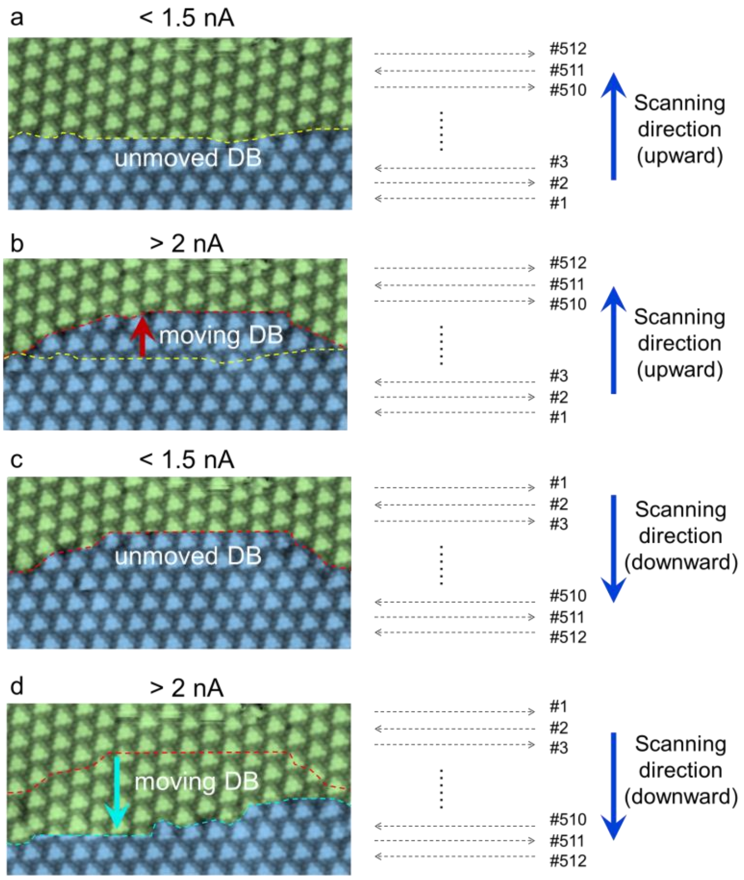

Supplementary Fig. 2. Time-lapse STM images showing the movement of the chiral CDW domain boundary. The DB movement is controlled by scanning direction and current setpoint.

We managed to control the movement direction of the DB by using different current setpoint values for the upward and downward scan. When the upward scan is performed with 2 nA, the DB moves up (from Fig. S2a to Fig. S2b); while the downward scan is performed with a current less than 1.5 nA, the DB remains unchanged in Fig. S2c. When the downward scan is performed with a current larger than 2 nA, the DB move downward (from Fig. S2c to Fig. S2d). This is like a broom-wiping process, and it may be related to a mechanical effect in combination with the electron tunneling effect. Thus, these measurements indicate that the DB movement can be triggered by a larger tunneling current (also demonstrated in Fig. 2), and the DB movement direction can be controlled with the tip scanning direction and current setpoint.

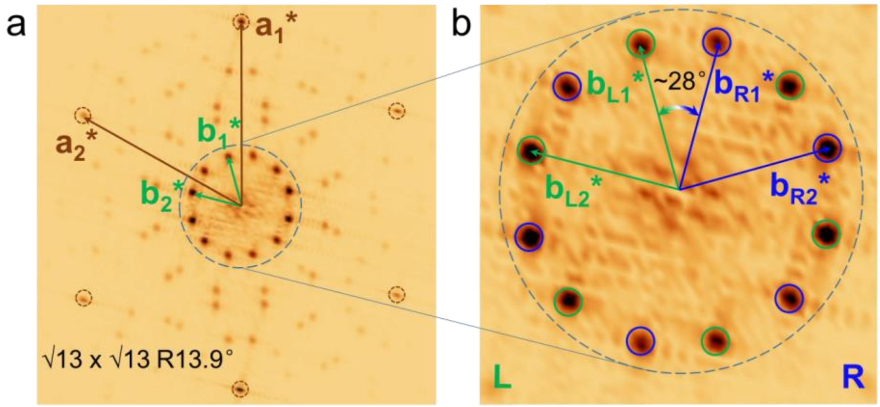

Supplementary Fig. 3. Fast Fourier transform (FFT) of chiral CDW domains. **a**, FFT images of the atomic STM images with two chiral CDW domains, showing that there is only one set of atomic Se lattices (the basis vectors $\mathbf{a}_1$* and $\mathbf{a}_2$* of in reciprocal space indicated by the long brown arrows) and two sets of CDW superlattices with mirror symmetry (basis vectors $\mathbf{b}_{L1}$* and $\mathbf{b}_{L2}$* from L domain, and $\mathbf{b}_{R1}$* and $\mathbf{b}_{R2}$* from the R domain, indicated by the short green arrows, respectively). For a simple visualization, the FFT images were rotated by 30 ° from the raw image. **b**, The magnified image of the area enclosed by the dashed circle in (**a**). The spots of the L and R chiral CDW domains are represented by green and blue circles, respectively.

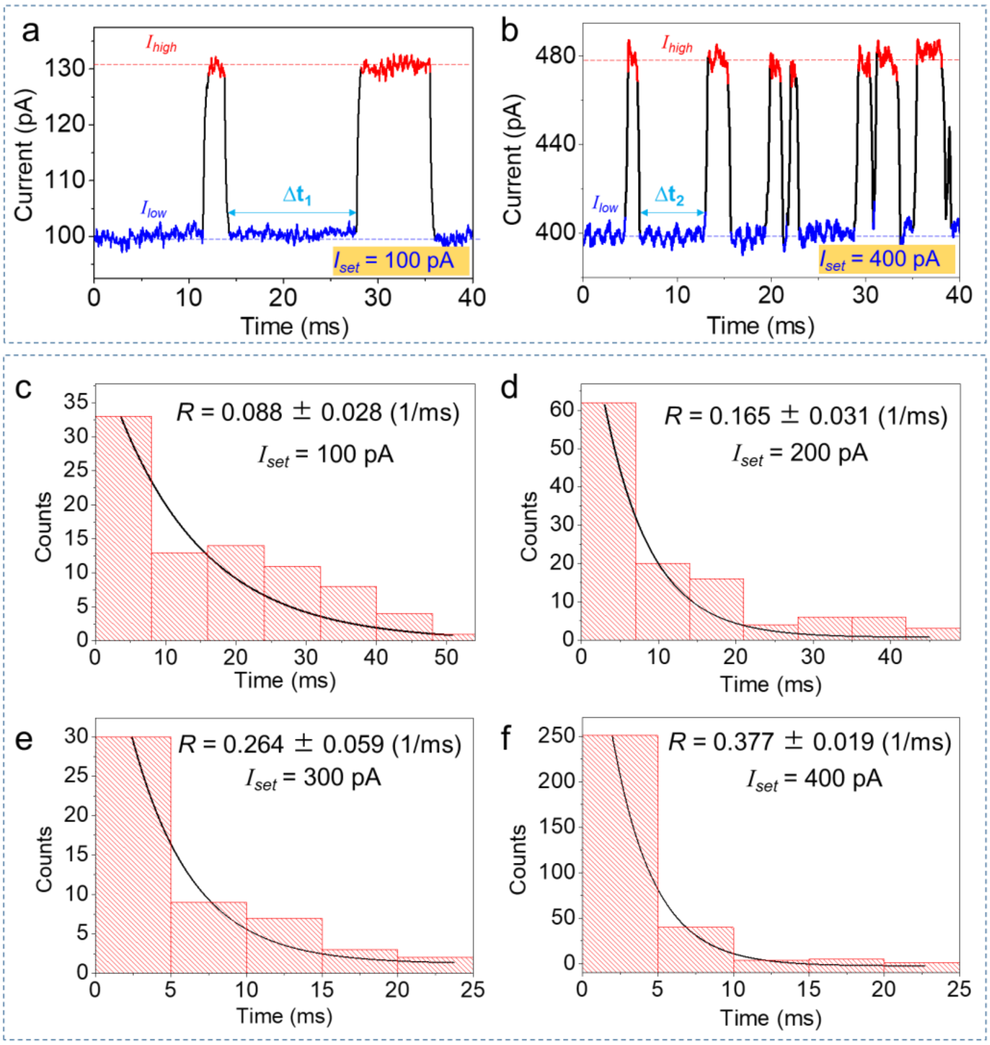

Supplementary Fig. 4. The dynamic characterization of the chiral domain transition. a and **b**, *I*-*t* curves with different current setpoints. Δt_1 and Δt_2 are the residence time of the SOD in the given chiral domains. **c-f**, Statistical analysis of the residence time distribution. The count of chiral CDW switching events (detected by I_{low} and I_{high}, when the SOD is "away from" and "right below" the STM tip) as a function of the residence time exhibits an exponential decay. The chiral CDW switching rate was determined by an exponential fit (black curve).

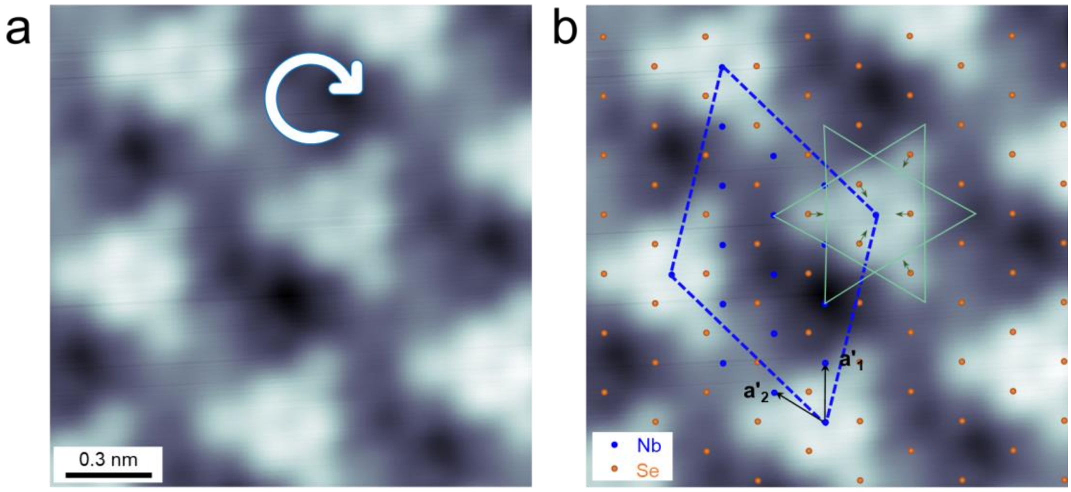

Supplementary Fig. 5. Tiny contraction (~30 pm) of the Se atomic lattice in the CDW superlattice. a, The atomic resolution STM image of several SOD clusters. **b**, The array of the orange balls represents the top-layer Se atom lattice, without considering the SOD contraction, the blue rhombus represents the CDW unit cell, and the green hexagram star represents the SOD cluster. The positions of the Se atoms are marked with orange balls. The positions of the Nb atoms are marked with blue balls.

As known by the cases of 1T-TaS_2, $TaSe_2$, and $NbSe_2$, the Star of David (SOD) is formed due to the contraction of the 12 outer Nb atoms toward the central Nb atom. The lateral contraction distance is approximately 20 pm[1]. It also induces a vertical shift of the Se atoms, which can be imaged by STM (triangular SOD) as the tunneling current is exponentially dependent on the tip–sample distance.

To reveal the contraction of the top-layer Se lattice before and after the SOD CDW formation, we overlay the model for the Se atomic lattice without considering the SOD contraction onto the STM image (Fig. S5). The construction of the Se atomic lattice consists of the following steps.

In the first step, the center position of the SOD cluster is marked (the green hexagram star) in the atomic-resolution STM image, which is the position of the central Nb atom in each SOD. It is well known that the other 12 Nb atoms contract toward the central atom, so that the center remains immobile.

In the second step, the center points of four neighboring SOD clusters are

connected to form a rhombus, which represents the unit cell of the SOD CDW. The positions of the Nb atoms are marked with blue balls within the rhombus according to the ($\sqrt{13} \times \sqrt{13}$)R13.9° superstructure relationship.

In the third step, the positions of the Se atoms are marked with orange balls, each of which is at the center of three adjacent Nb atoms. The resulting orange ball lattice represents the Se lattice without considering SOD contraction. The position difference between the centers of the Se atom (the white round protrusion) in the STM image and orange balls represents the SOD contraction.

In general, each orange ball well matches the center of the Se atom. The contraction is on the order of the diameter of the orange ball (~30 pm), which is at the lower limit of the STM resolution. This tiny distortion is challenging to detect and is not considered as a lattice defect in our discussion throughout this paper.

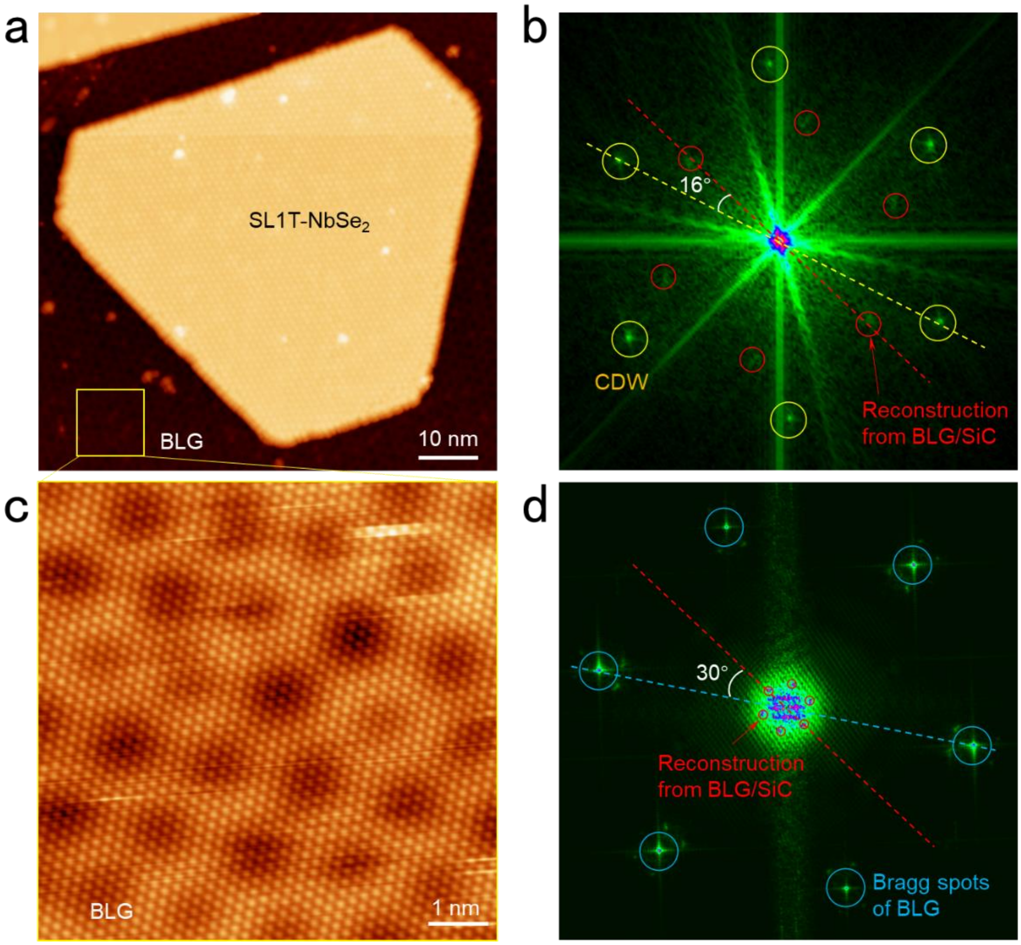

Supplementary Fig. 6. Additional Bragg spots from BLG by including wide area. (a) STM image of SL 1T-$NbSe_2$ of one chirality on BLG. (b) FFT of (a), showing the spots of the reconstruction from BLG/SiC and the CDW patterns of $NbSe_2$. (c) STM image of the BLG/SiC(0001) with carbon atomic lattice and the reconstruction. (d) FFT of (c), showing the spots of Bragg lattice of BLG and the reconstruction of BLG/SiC. The Bragg spots have a rotation of 14° (= 30° − 16°) with respect to those of CDW. Scanning parameters: a, -1.5 V, 20 pA; c, -1.5 V, 100 pA.

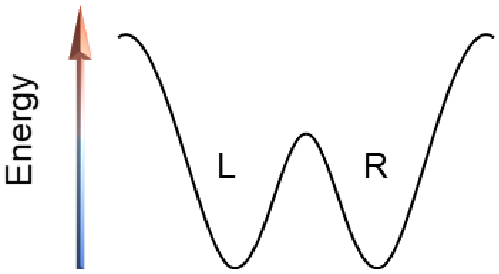

Supplementary Fig. 7. The energy barrier model of the L and R chirality.

As for the concern why not a larger disorder area at the DB or one chirality outcompeting the other, we think there are two possible reasons: One is the similar energy barrier of both L and R chiral domains, as shown by the “energy barrier model” in Fig. S7. The relatively stable SOD pattern prefers to maintain the narrow domain boundary. The other is the weak interface interaction of the monolayer $NbSe_2$ and the supporting graphene, showing no clear influence on the chirality formation and switching of the entire $NbSe_2$ domain, thus preserving the degeneracy of the L/R chiralities.

Movie S1.

Dynamic example of the DB movement of the chiral CDW superlattice.

References

1. Kikuchi, A. & Tsukada, M. Theory of STM Images of CDW in Transition-Metal Dichalcogenides. *Surf. Sci.* **326**, 195-207 (1995).
