# Supplementary material for: Atomic-scale visualization of chiral charge density wave superlattices and their reversible switching
Source: Nat Commun. 2022 Apr 5;13:1843. doi: 10.1038/s41467-022-29548-2 (PMC8983771; doi:10.1038/s41467-022-29548-2)
Supplement: Supplementary file 2 — Description of Additional Supplementary Files [file 41467_2022_29548_MOESM2_ESM.pdf]

Description of Additional Supplementary Files

File Name: Supplementary Movie 1
Description: Dynamic example of the DB movement of the chiral CDW superlattice.
