# Supplementary figures and images for: Atomic-scale visualization of chiral charge density wave superlattices and their reversible switching
Source: Nat Commun. 2022 Apr 5;13:1843. doi: 10.1038/s41467-022-29548-2 (PMC8983771; doi:10.1038/s41467-022-29548-2)

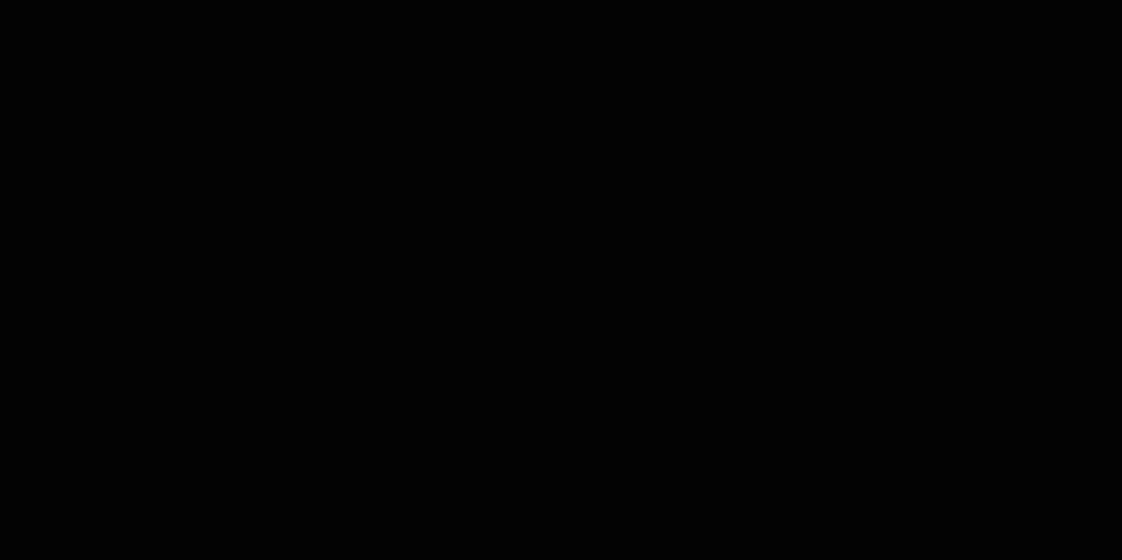

Supplement: Supplementary file 3 — Supplementary Movie 1 [file 41467_2022_29548_MOESM3_ESM.gif]
